# High-Throughput Study on Nanoindentation Deformation of Al-Mg-Si Alloys

**DOI:** 10.3390/ma18153663

**Published:** 2025-08-04

**Authors:** Tong Shen, Guanglong Xu, Fuwen Chen, Shuaishuai Zhu, Yuwen Cui

**Affiliations:** 1 Sino-Spain Joint Laboratory on Biomedical Materials (S2LBM), College of Materials Science and Engineering, Nanjing Tech University, Nanjing 211816, China; 202262103003@njtech.edu.cn (T.S.); fuwenchen@njtech.edu.cn (F.C.); 2 School of Materials Science and Engineering, Nanjing Institute of Technology, Nanjing 211167, China; zhuss@njit.edu.cn

**Keywords:** kinetic diffusion multiple, Al-Mg-Si alloy, nanoindentation, creep test, pop-in effect, indentation size effect, deformation mechanism

## Abstract

Al-Mg-Si (6XXX) series aluminum alloys are widely applied in aerospace and transportation industries. However, exploring how varying compositions affect alloy properties and deformation mechanisms is often time-consuming and labor-intensive due to the complexity of the multicomponent composition space and the diversity of processing and heat treatments. This study, inspired by the Materials Genome Initiative, employs high-throughput experimentation—specifically the kinetic diffusion multiple (KDM) method—to systematically investigate how the pop-in effect, indentation size effect (ISE), and creep behavior vary with the composition of Al-Mg-Si alloys at room temperature. To this end, a 6016/Al-3Si/Al-1.2Mg/Al KDM material was designed and fabricated. After diffusion annealing at 530 °C for 72 h, two junction areas were formed with compositional and microstructural gradients extending over more than one thousand micrometers. Subsequent solution treatment (530 °C for 30 min) and artificial aging (185 °C for 20 min) were applied to simulate industrial processing conditions. Comprehensive characterization using electron probe microanalysis (EPMA), nanoindentation with continuous stiffness measurement (CSM), and nanoindentation creep tests across these gradient regions revealed key insights. The results show that increasing Mg and Si content progressively suppresses the pop-in effect. When the alloy composition exceeds 1.0 wt.%, the pop-in events are nearly eliminated due to strong interactions between solute atoms and mobile dislocations. In addition, adjustments in the ISE enabled rapid evaluation of the strengthening contributions from Mg and Si in the microscale compositional array, demonstrating that the optimum strengthening occurs when the Mg-to-Si atomic ratio is approximately 1 under a fixed total alloy content. Furthermore, analysis of the creep stress exponent and activation volume indicated that dislocation motion is the dominant creep mechanism. Overall, this enhanced KDM method proves to be an effective conceptual tool for accelerating the study of composition–deformation relationships in Al-Mg-Si alloys.

## 1. Introduction

6XXX series aluminum alloys are extensively employed in engineering structures, including automotive and aerospace applications, owing to their excellent mechanical properties, machinability, corrosion resistance, weldability, and lightweight nature [1,2]. These alloys mainly contain magnesium (Mg) and silicon (Si), with Mg-Si precipitates forming during aging to provide precipitation strengthening. To fully leverage their advantages in structural applications, it is crucial to comprehensively evaluate mechanical properties—such as hardness and creep stress exponent—and to deepen the understanding of their deformation mechanisms.

The contents of Mg and Si critically govern the mechanical performance of Al-Mg-Si alloys. Excess Si promotes the precipitation of β″ phase, resulting in a finer and more uniformly distributed β″ precipitate structure that enhances age-hardening strength. However, this improvement comes at the cost of reduced toughness and corrosion resistance [3]. In contrast, excess Mg enhances corrosion resistance and weldability but tends to accelerate the growth and coarsening of the β″ phase [4,5]. The complex interactions between Mg and Si significantly affect the alloy’s microstructure and deformation behavior, complicating performance testing and optimization within a vast and intricate compositional space that remains largely unexplored. Moreover, creep damage during prolonged service increases demands on the structural efficiency and lifespan of Al-Mg-Si alloys. Traditional creep testing methods [6], being time-consuming, are insufficient for evaluating and optimizing alloy performance under such conditions.

Assessing the mechanical properties of existing and novel Al-Mg-Si alloy compositions using conventional trial-and-error methods is both time-consuming and costly [7,8]. High-throughput (HT) experimental approaches—central to the Materials Genome Initiative combined with integrated computational materials engineering—are increasingly adopted to accelerate the development of composition–process–structure–property–performance relationships for material design and optimization. For instance, the kinetic diffusion multiple (KDM) technique creates continuous compositional–microstructural gradients within a single sample [9,10], enabling rapid exploration of composition–property correlations within multicomponent alloy systems [11]. This approach offers a novel technological pathway for evaluating mechanical properties and uncovering deformation mechanisms throughout various complex alloys [12,13,14].

Nanoindentation is a highly efficient micro-mechanical testing method, particularly suitable for high-throughput experiments that measure hardness, creep behavior, and deformation phenomena within a short timespan [15,16,17]. For instance, Wang et al. investigated the influence of Ga content on the creep resistance of Mg-Ga alloys using a liquid–solid diffusion couple [18], while Mao et al. demonstrated that increasing Zn content improved the creep resistance of Mg-Zn alloys [7]. Currently, the diffusion multiple technique has been successfully applied to investigate the creep properties of Mg alloys. However, there have been no reports on the preparation of Al-Mg-Si alloys using KDM. There remains a significant gap in the systematic exploration of creep properties in multicomponent Al-Mg-Si alloy systems using KDM. Moreover, studies focusing on the indentation size effect (ISE) and pop-in phenomena in Al-Mg-Si alloys prepared by KDM are currently absent.

This study employs the KDM method as its core technique, performing solution and aging heat treatments on Al-Mg-Si alloys to simulate practical production conditions. Electron probe microanalysis (EPMA) and nanoindentation are integrated for mechanical property testing on a KDM designed for covering the Al-Mg-Si alloys. It systematically examines how the ISE, pop-in effect, and creep behavior of Al-Mg-Si alloys vary with the Mg and Si contents in a high-throughput manner. The objectives are to uncover the deformation mechanisms of Al-Mg-Si alloys with varying compositions by scanning over the composition gradients during nanoindentation tests and to provide valuable insights and guidelines for designing these alloys, thereby accelerating both the optimization of existing alloys and the discovery of new ones.

## 2. Experimental Procedure

### 2.1. Fabrication of KDM

**Figure 1:**
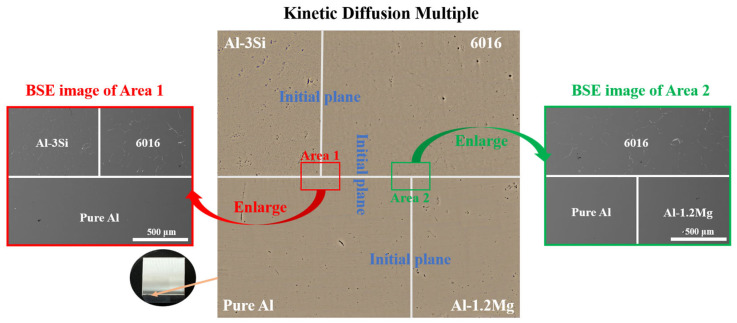
6016/Al-3Si/Al-1.2Mg/Al quadruple KDM.

Four endmember blocks—Al-1.2Mg (wt.%), Al-3Si (wt.%), 6016 alloy, and high-purity aluminum (>99.99 wt.%)—were selected based on compositional design. These blocks were homogenized, then cut into smaller pieces using an electrical discharge wire cutting machine, with their contact surfaces polished. The four smaller blocks were diffusion-bonded in a vacuum of 6 × 10^−2^ Pa at 540 °C, successfully fabricating the KDM shown in Figure 1. The KDM was then held at 530 °C for 72 h and quenched in cold water, promoting the formation of junction areas Area1# and Area2# with diffusion penetration exceeding one thousand micrometers. Subsequent solution and aging heat treatments (530 °C for 30 min followed by 185 °C for 20 min) were applied to simulate actual production conditions. Finally, the KDM was polished, and Vickers hardness indentations were made at Area1# and Area2# to enable subsequent compositional and nanoindentation testing.

### 2.2. Composition Tests

Electron microprobe analysis (EMPA) using a JEOL JAX-8900 microscope (JEOL Ltd, Tokyo, Japan), operating at 20 kV with a beam current of 20 × 10^−8^ A, to determine the local chemical compositions within the diffusion zones of the KDM. Only EPMA data with total weight percentages between 99% and 101% were retained for this study. Nanoindentation CSM and creep testing were conducted at selected points (termed as micro-alloy, i.e., MA) on the KDM. The atomic compositions of these MAs are presented in Table 1, where MAs with varying compositions are labeled accordingly.

### 2.3. Nanoindentation Tests

The mechanical properties of MAs on the KDM, which were mechanically polished to a mirror finish, were measured using a Nano Indenter G200 (MTS, Agilent, Santa Clara, CA, USA) equipped with a standard Berkovich diamond indenter at room temperature. Two testing methods were applied, constant strain rate (CSR) and constant loading rate (CLR), with each condition repeated three times per MA to obtain average values. Prior to nanoindentation, the indenter tip was calibrated with a fused silica standard reference sample.

The continuous stiffness measurement (CSM) method recorded hardness (*H*), modulus (*E*), and contact stiffness (*S*) as functions of indenter displacement up to 1500 nm under a strain rate of 0.05 s^−1^. *S* was monitored continuously by adding a sinusoidal signal (45 Hz, 2 nm amplitude) to a DC load, while thermal drift was regulated and kept below 0.05 nm/s.

Nanoindentation creep experiments were performed under load control, applying a peak load of 15 mN and a loading rate of 0.15 μN/s. The maximum indentation depth depended on the material’s response, particularly its creep characteristics. During the creep test, the load was held constant at peak value for 900 s. After the dwell period, the indenter was reduced to 10% of the peak load and maintained for 120 s to correct for thermal drift.

## 3. Equations Used in Nanoindentation Measurements

### 3.1. Hardness and Elastic Modulus Measurements

Oliver and Pharr [19] proposed a technique to determine the indentation hardness of materials. In self-similar indentations, such as Berkovich indenters, the contact area *A* can be expressed as a function of the indentation displacement *h* on the specimen’s surface [20,21]:

(1) $$A=24.56\times h^{2}$$

In nanoindentation testing, the hardness *H* is defined as

(2) $$H=\frac{P}{A}$$

where *P* represents the applied load. The Young’s modulus *E* of the material can likewise be calculated using the following equation during the indentation test [22]:

(3) $$\frac{1}{E_{r}}=\frac{1-v_{i}^{2}}{E_{i}}+\frac{1-v^{2}}{E}$$

where *E_i_* denotes the indenter’s modulus (*E_i_* = 1140 GPa); *v*_i_ is the Poisson’s ratio of the diamond indenter (*v_i_* = 0.07) [8,23]; *v* represents the Poisson’s ratio of the tested material; and *E*_r_ is the reduced modulus, which depends on the contact area *A* and contact stiffness *S*.

(4) $$S=\frac{dP}{dh}=\frac{2}{\sqrt{\pi }}Er\sqrt{A}$$

where *S* denotes the slope of the initial unloading segment. In the CSM method, *S* can be monitored continuously throughout the loading stage of an indentation test.

### 3.2. Nanoindentation Creep Measurement

By conducting nanoindentation creep experiments, the stress exponents of different components can be calculated. During the creep stage, according to the exponential creep law, the relationship between the strain rate $\dot{\varepsilon }$ and the equivalent stress σ is established based on the power law equation:

(5) $$\dot{\varepsilon }=A_{0}\sigma^{n}$$

where *A*_0_ serves as the material constant and *n* represents the creep stress exponent. For the self-similar indentation like Berkovich indenter, the relationship between σ and *H* is as follows:

(6) $$\sigma =k_{1}H$$

where *k*_1_ is a constant. Usually, the value of *k*_1_ is 1/3. Strain rate $\dot{\varepsilon }$ can be defined as

(7) $$\dot{\varepsilon }=\frac{1}{h}\frac{dh}{dt}=\frac{\dot{h}}{h}$$

where $\dot{h}$ represents the displacement rate, obtained by empirically fitting the creep displacement–holding time data collected at a constant load.

(8) $$h(t)=h_{0}+a{\left(t-t_{0}\right)}^{p}+kt$$

where *h*_0_, *a*, *t*_0_, *p,* and *k* are the fitting parameters, and *h*, *t* are the indenter displacement and time of the creep process starting point.

According to Equations (5)–(8), a new equation can be formulated as follows:

(9) $$\ln\dot{\varepsilon }=n\ln(\sigma )+\ln C$$

where *C* is a constant. The creep stress exponent *n* can be calculated from the slope of ln$\dot{\varepsilon }$ versus lnσ curves [24]. After completing the nanoindentation creep test, the activation volume V* is determined using the subsequent equation [25]:

(10) $$V*=\sqrt{3}KT(\frac{\partial \ln\dot{\varepsilon }}{\partial \sigma })=\frac{3\sqrt{3}KTn}{H}$$

where *K* denotes Boltzmann constant (*K* = 1.38 × 10^−23^ J/K), and *T* represents the absolute temperature.

## 4. Results

### 4.1. Constant Strain Rate Tests

**Figure 2:**
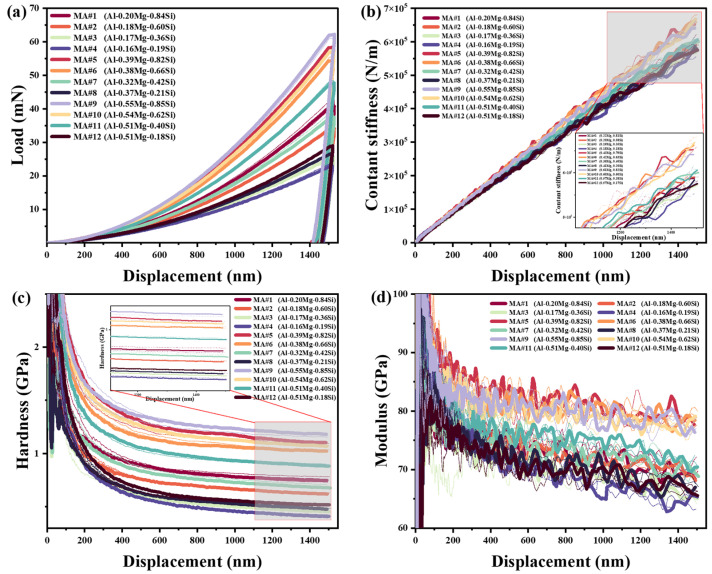
Indenter displacement dependence of (**a**) load, (**b**) contact stiffness, (**c**) hardness, and (**d**) modulus.

The CSM nanoindentation test results are presented in Figure 2. Three tests were performed for each MA, with the dashed lines of the same color representing the repeated tests for each MA. As shown, the three tests per MA demonstrate excellent reproducibility. Figure 2a shows the load–displacement curves, where MA#9 (Al-0.55Mg-0.85Si, wt.%) exhibits the highest load at a displacement of 1500 nm. The load values for MA#5 (Al-0.39Mg-0.82Si, wt.%) and MA#10 (Al-0.54Mg-0.62Si, wt.%) are slightly lower but close to that of MA#9. As shown in Table 2, the load values for the remaining MAs decrease with decreasing alloy content. At constant alloy content, the loads are higher when the Mg/Si ratio approaches 1.

As shown in Figure 2b, contact stiffness *S* increases proportionally with displacement. Consequently, according to Equation (4), the reduced modulus *E_r_* remains constant for each MA. Figure 2c illustrates the variation in hardness with displacement: hardness initially decreases rapidly as displacement increases, but beyond approximately 1000 nm, the rate of decrease slows significantly. This phenomenon is known as the indentation size effect (ISE). The trend of the modulus with displacement, shown in Figure 2d, closely follows that of hardness, with the average indentation modulus nearly constant once displacement exceeds about 1000 nm. Based on these relationships, MAs #5, #6, #9, and #10 exhibit higher contact stiffness, hardness, and modulus values.

### 4.2. Nanoindentation Creep Tests

**Figure 3:**
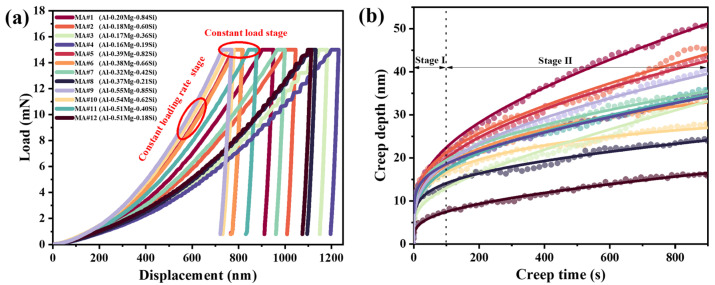
Nanoindentation creep results for MAs with different compositions: (**a**) load–displacement curves; (**b**) creep displacement-time curves.

Creep studies were conducted on different MA compositions under a constant load of 15 mN. Figure 3a shows representative load–displacement curves for various compositions. Due to differences in MA composition, the displacement required to reach the set load varies. MA#9 required the least displacement to reach the target load, followed by MA#10. The plateau in Figure 3a corresponds to the constant load-holding stage.

Figure 3b presents displacement curves plotted against creep time, with all initial displacements set to zero for direct comparison. The displacement–time curve consists of two stages, with no rupture occurring due to the localized load. Stage I represents the transient creep stage, characterized by an initially rapid increase in creep displacement that gradually slows as creep resistance or strain hardening develops. Following this, Stage II begins—the steady-state creep stage—where displacement increases at an approximately constant rate. Additionally, Figure 3b shows that MA#1 exhibits the largest creep displacement during the specified time, while MA#12 shows the smallest displacement over the same period.

**Figure 4:**
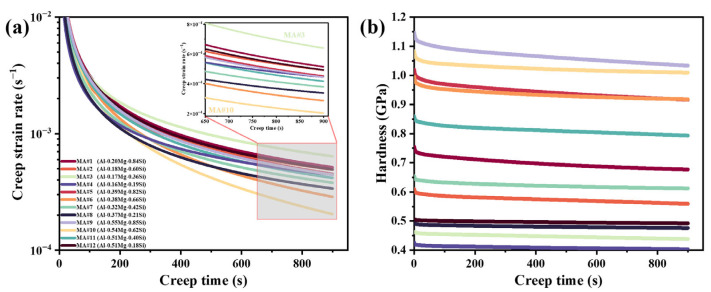
Nanoindentation creep results for MAs with different compositions: (**a**) strain rate–time curves; (**b**) hardness–time curves.

As the indenter advances into the MA material throughout the creep stage, the strain rate and hardness variations with creep time were calculated using Equations (1), (2), (7) and (8). Figure 4a shows the relationship between creep strain rate and creep time for different compositional MAs. At the initial stage of creep, the strain rate for all MAs decreases rapidly with increasing creep time, then gradually slows before stabilizing. MA#3 exhibits the highest strain rate during this stage, while MA#10 shows the lowest.

Figure 4b illustrates the variation in creep hardness with creep time for each MA. Hardness initially decreases rapidly as creep time increases, then the rate of decrease slows after approximately 50 s. Combined with the data in Figure 3b, this suggests that the ISE also occurs during the creep stage. MA#9 shows the highest hardness throughout the creep stage, whereas MA#4 exhibits the lowest. The initial hardness values *H*_ini_ at the start of the creep stage for each MA are summarized in Table 3.

**Figure 5:**
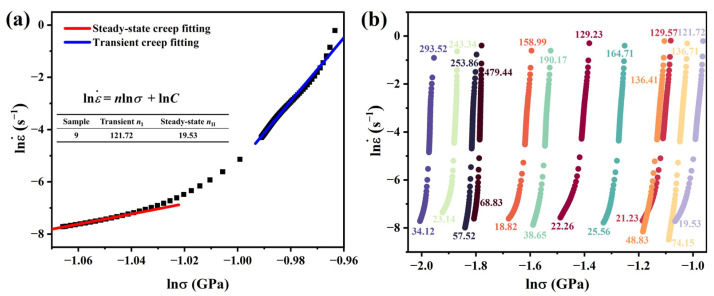
Creep stress exponent at different compositional MAs: (**a**) MA#9; (**b**) all MAs.

Taking MA#9 as an example, the creep stress exponents *n*_I_ and *n*_II_ for Stage I and Stage II were calculated. Figure 5a illustrates the calculation process. According to the theory described earlier, the stress exponent *n* is obtained from the gradient of the log$\dot{\varepsilon }$-vs.-logσ plot. These plots typically exhibit two distinct linear segments: the linear high-hardness region (LHHR) associated with Stage I and the linear low-hardness region (LLHR) linked to Stage II.

Figure 5b presents the log–log relationship between strain rate and stress during the creep stage for all selected compositional MAs. The values above each curve represent *n*_I_ for Stage I, while the values below correspond to *n*_II_ for Stage II. In Stage II, MA#9 exhibits the lowest stress exponent at 19.53, whereas MA#10 shows the highest at 74.15.

## 5. Discussion

### 5.1. Pop-In Effect During Loading

**Figure 6:**
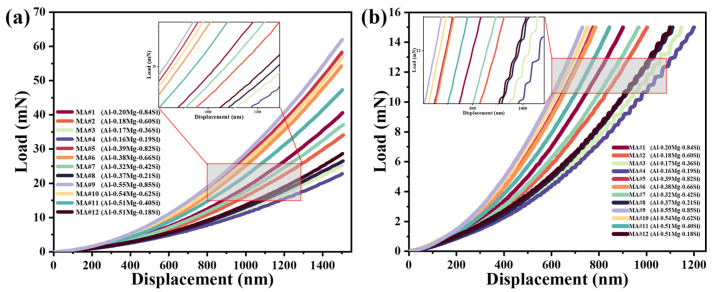
Load–displacement curves of the MAs at the loading stage with different compositions: (**a**) CSR; (**b**) CLR.

The load–displacement (P–h) curves from the loading cycle were obtained for various MAs under constant strain rate (CSR) and constant loading rate (CLR) conditions. Figure 6a presents the P-h curves under CSR mode, while Figure 6b shows these under CLR mode. In both modes, low-alloy MAs (#3, #4, #8, #12; Mg + Si < 0.7 wt.%) exhibit distinct serrated flow marked by “pop-in” occurrences, referred to as the Portevin–Le Chatelier (PLC) effect [26,27]. This serrated behavior during loading is attributed to dislocation nucleation coupled with dynamic strain aging (DSA), driven by interactions between migrating solute atoms and mobile dislocations [28].

In general, in annealed metals with low dislocation density, the early indentation response is entirely elastic and fully reversible [22,29]; with increasing load, the material yields to irreversible plastic deformation, where dislocation nucleation and movement begin [30]. According to the theoretical elastic response described by the Hertzian theory [31], the beginning of plastic deformation in nanoindentation can be identified as the first deviation of the experimental load–displacement curve from the elastic Hertzian curve.

During nanoindentation, dislocations encountering obstacles such as forest dislocations or precipitates become temporarily pinned. During this pinning, solute atoms like Mg and Si diffuse to low-energy sites, further reinforcing these obstacles against dislocation motion [32]. The presence of solute atoms restricts dislocation movement, causing temporary hardening of the material. As the stress from the indenter displacement increases, dislocations overcome the obstacles, detach from solute atoms, and continue to move, which reduces the force needed for further deformation [33]. However, as dislocations progress, they soon encounter new pinning points and become pinned again, repeating this process [28]. Comparable behavior have been reported in various solid solution alloys and metallic glasses under indentation [34,35,36]. This phenomenon is ascribed to the repeated pinning and unpinning of dislocations, coupled with their detachment from solute atoms during loading.

It is also worth noting that high-alloy MAs (#5, #9, #10; Mg + Si > 1.0 wt.%) show suppressed pop-in (<2 serrations per curve), attributable to β″ precipitates acting as dislocation barriers. Crucially, MA#9 displays near-zero pop-in despite moderate solute content (Figure 6a), suggesting precipitate-dominated hardening outweighs solute drag. This gradient-enabled observation reveals a critical Mg/Si atomic ratio threshold (~0.7–1.0) where precipitate effects override DSA. Furthermore, with less alloying elements, there are fewer obstacles, allowing individual shear bands to extend sufficiently to accommodate strain and propagate within the material. However, as alloy content rises, the increased number of obstacles prevents individual shear bands from accommodating strain, promoting the development and extension of multiple shear bands instead [37,38]. This inhibits strain accommodation and thus suppresses the serrated flow behavior.

### 5.2. Indentation Size Effect on Hardness

The CSM and creep tests indicate that alloy hardness decreases with increasing indentation depth, exhibiting a pronounced indentation size effect (ISE). As illustrated in Figure 2c, hardness measurements for indentations shallower than 100 nm exhibit increased scatter. This variability is attributed to surface-related factors and artifacts such as deformation layers, surface oxides, and a blunted indenter tip [17,20,39].

**Figure 7:**
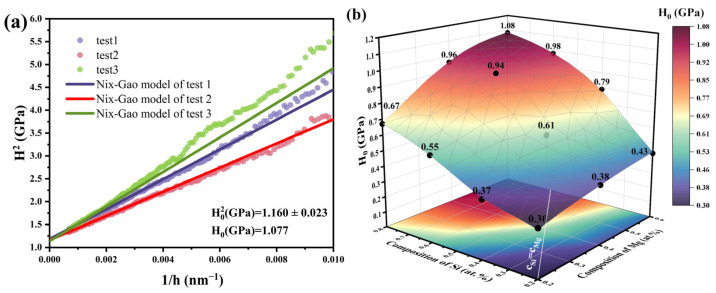
(**a**) Nix-Gao model fitting of depth-dependent hardness for MA#9. (**b**) Distribution of Nix-Gao model fitting hardness values for all 12 MAs.

The presence of ISE during nanoindentation complicates the accurate extraction of hardness. The Nix-Gao model [40], which incorporates the influence of geometrically necessary dislocations (GNDs) on measured hardness [41], is widely used to correct the ISE. This model allows for the estimation of the bulk-equivalent hardness *H*_0_ from raw nanoindentation data using the following classical equation:

(11) $$H=H_{0}\sqrt{1+\frac{h^{*}}{h_{c}}}$$

where *H* is the measured hardness at indentation depth, and h^*^ is a characteristic length. Using MA#9 as an example, the bulk-equivalent hardness *H*_0_ was determined via the Nix-Gao model, as shown in Figure 7a. In this study, hardness tends to stabilize when the displacement exceeds 500 nm, so the model was fitted to data at depths greater than 500 nm, achieving an *R*^2^ value above 0.99.

According to the Nix-Gao model [40], the flow stress in crystalline materials arises from two dislocation density components: geometrically necessary dislocations (GNDs), which originate from lattice curvature or strain gradients, and statistically stored dislocations (SSDs), produced by homogeneous plastic strain. The contribution of GNDs to hardness decreases continuously with increasing indentation depth, while the SSD contribution is primarily influenced by the microstructural features of the material.

For the KDM alloy, grains are coarse due to prolonged high-temperature interdiffusion annealing. In coarse-grained (CG) metals, plasticity is primarily governed by the multiplication and motion of dislocations from intragranular sources [42]. Once yielding occurs, the SSD density reaches a steady-state plastic saturation [43]. Therefore, the pronounced ISE in KDM is mainly controlled by GND density, with SSD density exerting only a minor influence on hardness variation at larger indentation depths [44].

Figure 7b presents the distribution of *H*_0_ values for all 12 MAs. It is evident that *H*_0_ increases continuously with rising alloy content. Compared to Mg, an increase in Si content results in a faster rise in *H*_0_, due to the larger atomic size mismatch between Si and Al atoms—causing greater lattice strain—than that between Mg and Al atoms [45]. Consequently, the solid solution strengthening effect of Si is stronger than that of Mg. MA#10 (Mg/Si atomic ratio = 1) achieves a higher *H*_0_ (0.98 GPa), while MA#5 (Mg/Si atomic ratio = 0.54) underperforms (*H*_0_ = 0.96 GPa) despite higher Si (0.79 at.%)—contradicting classical solid solution models. Moreover, as indicated by the white line in the figure, when the total alloying content remains constant and Mg and Si contents are approximately balanced, *H*_0_ reaches its maximum. Synergistic hardening at Mg/Si ≈ 1 correlates with β″ nucleation efficiency, implying precipitate density governs bulk hardness more than solute content alone.

### 5.3. Indentation Creep Mechanisms

**Figure 8:**
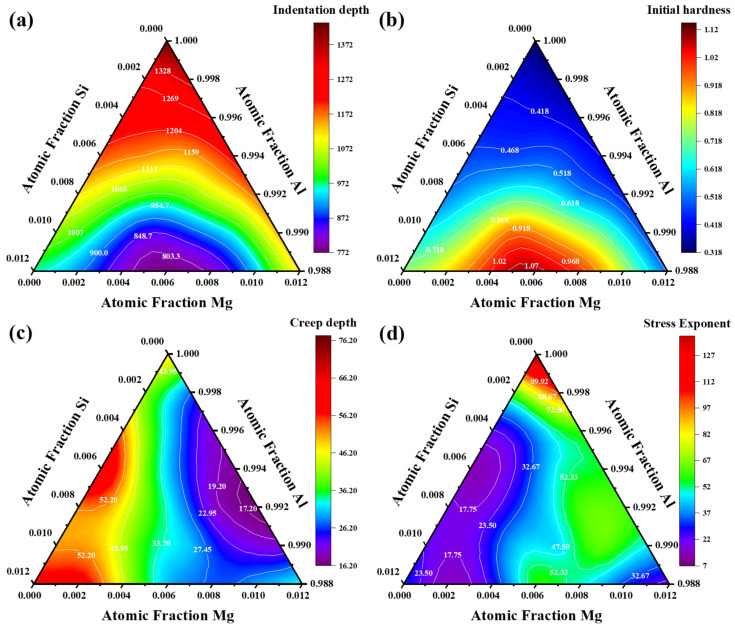
Distribution of mechanical properties of the Al-Mg-Si alloy with different compositions: (**a**) *h*_max_, (**b**) *H*_Ini_, (**c**) *h*_creep_, and (**d**) *n*_II_.

The relationships between composition, maximum indentation depth *h*_max_, initial hardness *H*_Ini_ at the beginning of the creep test, creep depth *h*_creep_, and stress exponent *n*_II_ during the creep test are shown in Figure 8. As illustrated in Figure 8a, throughout the creep, *h*_max_ decreases continuously with increasing total Mg and Si content. Combining this with Figure 8b reveals an inverse relationship between *h*_max_ and *H*_Ini_. The initial hardness *H*_Ini_ depends on MA composition. As discussed in Section 5.2, increasing alloy content enhances solid solution strengthening. Additionally, when the total alloy content is constant and the Mg/Si atomic ratio approaches 1, the MA exhibits its highest hardness. This is also because the GP zones and β″ phase have the largest driving force to precipitate during aging in the alloys with a Mg/Si ratio close to 1 [2].

In nanoindentation creep analysis, the stress exponent *n* correlates with the dominant deformation mechanism [46]. When *n* is less than 1, diffusion creep predominates; grain boundary sliding creep corresponds to *n* between 1 and 2. In this study, creep experiments were conducted inside individual grains at peak load—thanks to the coarse grain structure—to reduce grain boundary influences. Once the stress exponent *n* surpasses 3, dislocation movement typically governs the creep response [47]. As shown in Figure 3a and Figure 8d, all creep experiments were conducted at depths greater than 500 nm, well within an effective diffusion penetration, implying that creep occurs primarily via dislocation motion. High-throughput nanoindentation creep tests reveal that dislocation motion dominates the deformation process across all MAs, as evidenced by stress exponents consistently exceeding *n* > 3, confirming dislocation-mediated mechanisms [48]. The compositional gradients in the KDM sample offer unprecedented details about how minor elemental variations alter deformation pathways. Specifically, the dramatic divergence in steady-state stress exponents (*n*_II_) between MA#9 and MA#10 demonstrates a critical compositional threshold: MA#9 exhibits dislocation glide-controlled creep with *n*_II_ = 19.5, while MA#10 shows cross-slip dominated deformation with *n*_II_ = 74.2—a 3.8-fold difference at nearly identical solute content.

In Stage I, the transient creep stage, the effective stress and strain rate are high. Strain rates start very high and then decrease as the indenter penetrates further into the KDM. These high strain rates cause significantly increased contact stresses, resulting in a high dislocation nucleation rate [17]. Consequently, a large density of GNDs is generated rapidly, which impedes dislocation motion and causes interactions among dislocations. As the indenter penetrates deeper, the strain rate decreases, leading to a lower dislocation nucleation rate. This explains why the stress exponent in Stage I *n*_I_ is much larger than that in Stage II *n*_II_.

Studies have shown that the stress exponent *n*_I_ in Stage I has no correlation with penetration depth [49], whereas in Stage II, the stress exponent *n*_II_ exhibits a strong size dependence on the indentation depth, particularly at depths below 100 nm [50,51]. In this study, all indentations were performed under identical loading conditions, with creep depths well above 500 nm. Therefore, size effects on the stress exponent can be excluded from our experimental results.

As shown in Figure 8c,d, the compositional MA with the largest creep depth *h*_creep_ has the smallest *n*_II_. This is because the magnitude of *n* depends on the balance between dislocation generation and annihilation. A higher *n* value generally indicates more dislocations are generated and involved during deformation [25]. A larger *h*_creep_ denotes increased plastic deformation, during which dislocations move with fewer hindrances and reduced entanglement. As a result, dislocations glide more easily, leading to a smaller *n*_II_.

When the MA composition approaches zero, *n*_II_ reaches its highest value, while the *n*_II_ values for the other MAs are more scattered. This variation is due to differences in the drag effect of solid solution atoms and precipitates on dislocations caused by compositional differences at a fixed load. Additionally, differences in indentation depth at the start of the creep process result in varying dislocation densities and types at different compositional MAs.

These dislocation structures are inherently unstable, and once the sample enters a stable plastic deformation regime, partial dislocations can annihilate through dislocation reactions [52]. Consequently, during creep, changes in the dominant dislocation motion mechanisms—such as dislocation glide or dislocation climb—cause the scatter observed in the creep stress exponents [53].

Moreover, we observed that the stress exponents in nanoindentation creep are higher than the typical values reported in the literature. This difference stems from the fact that conventional stress exponents are highly sensitive to external stress or hardness conditions [54]. In nanoindentation creep experiments, the applied stress (i.e., hardness) is significantly higher than in conventional creep tests, which usually operate below the yield strength. Consequently, higher strain exponents are expected in our experiments. Furthermore, high values of *n* have also been attributed to volumetric densification and dislocation pile-up mechanisms associated with creep [55].

**Figure 9:**
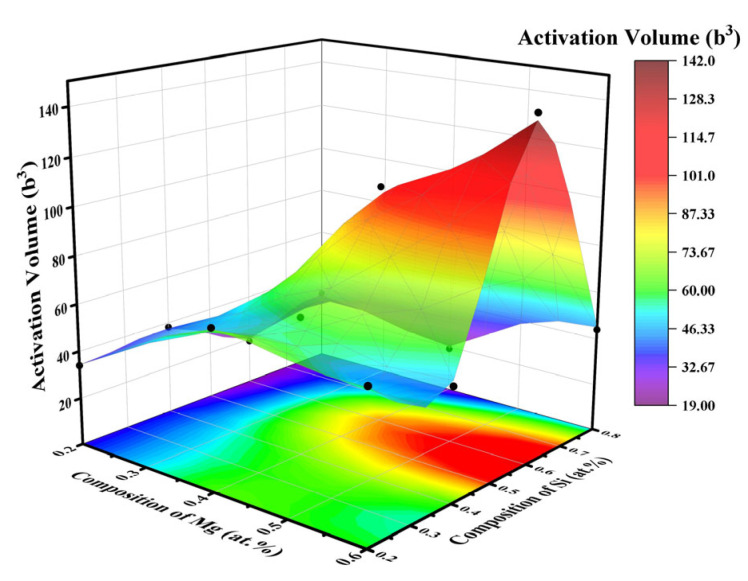
Distribution of activation volume for different MAs.

To further investigate the creep mechanism, the activation volume (V*) for the ternary alloy system was evaluated using Equation (10). The results are summarized in Figure 9 and Table 3, where b represents the Burgers vector for aluminum (b = 0.286 nm) [52]. The activation volume corresponds to the area traversed by dislocation segments in a single thermally activated event within crystalline materials whose plasticity is controlled by dislocation motion.

As shown in Figure 9, within the tested compositional range, the activation volume (V*) values range from 19 b^3^ to 142 b^3^. Low V* values below 100 b^3^ (e.g., MA#2 = 19 b^3^) indicate dense nanoscale barriers arising from solute atmospheres. In contrast, high V* values exceeding 100 b^3^ (e.g., MA#10 = 142 b^3^) reflect long-range obstacles originating from precipitate clusters [56,57,58]. Synergistic performance is achieved at Mg/Si atomic ratio≈ 0.75 (MA#9), where β″ precipitates optimize barrier density without excessively constraining dislocations. Consequently, MA#9 exhibits minimal creep depth, despite a moderate initial hardness (*H*_ini_ = 1.15 GPa). Furthermore, the alloy demonstrates a balanced work-hardening exponent (*n*_II_ = 19.53) and moderate activation volume (V* = 44b^3^), thereby imparting enhanced resistance against both dislocation glide and climb. Significantly, even at identical magnesium concentrations, distinctly different deformation mechanisms are observed, underscoring the critical influence of the Mg/Si ratio on dislocation interaction behaviors.

## 6. Conclusions

EPMA was used to establish different compositional MAs in the effective interdiffusion areas with gradients on the 6016/Al-3Si/Al-1.2Mg/Al kinetic diffusion multiple. Nanoindentation CSM and creep tests were carried out at room temperature to study the effect of composition on the pop-in effect, indentation size effect (ISE), and creep behavior of Al-Mg-Si alloys in a high throughput manner. The key findings are summarized below:

- Increased alloy content suppresses the occurrence and magnitude of pop-in events, indicating that higher alloying concentrations enhance obstacle density, thereby impeding dislocation motion. Although direct evidence of dislocation nucleation was not observed, the suppression of the pop-in effect can be reasonably attributed to hindered dislocation activity under higher solute concentrations. A critical Mg/Si atomic ratio threshold (~0.7–1.0) was identified, where precipitate hardening dominates over solute drag effects in suppressing pop-in.
- At larger indentation depths, the ISE is primarily governed by the density of GNDs, while the density of SSDs has minimal influence on hardness variations. Based on the Nix-Gao analysis, the corrected hardness *H*_0_ increases with Si content, suggesting that Si atoms contribute more significantly to strengthening than Mg atoms. Notably, under a constant total alloying level, an atomic Mg-to-Si ratio of approximately 1 yields the highest *H*_0_, indicating a potential synergistic effect at this composition.
- The stress exponent and activation volume suggest that dislocation motion dominates the steady-state creep stage. During the transient creep stage, high strain rates significantly increase contact stress, leading to an elevated dislocation nucleation rate and a high density of GNDs. Consequently, the *n*_I_ value is much larger than *n*_II_.

The reinforced KDM method has proven to be a powerful tool for investigating the relationship between composition and deformation mechanisms, facilitating the accelerated discovery and optimization of Al-Mg-Si alloys.

## Figures and Tables

**Table 1:** Composition of micro-alloys (MAs).

MA	1	2	3	4	5	6	7	8	9	10	11	12
Mg (wt.%)	0.20	0.18	0.17	0.16	0.39	0.38	0.32	0.37	0.55	0.54	0.51	0.51
Si (wt.%)	0.84	0.60	0.36	0.19	0.82	0.66	0.42	0.21	0.85	0.62	0.40	0.18
Mg (at.%)	0.22	0.20	0.19	0.18	0.43	0.42	0.36	0.41	0.61	0.60	0.57	0.57
Si (at.%)	0.81	0.58	0.35	0.18	0.79	0.63	0.40	0.20	0.82	0.60	0.38	0.17

**Table 2:** MA content vs. load values.

MA	Mg (wt.%)	Si (wt.%)	Mg + Si	Load (mN)
4	0.16	0.19	0.35	23.29 ± 0.41
3	0.17	0.36	0.53	26.06 ± 0.74
8	0.37	0.21	0.58	26.68 ± 0.31
12	0.51	0.18	0.69	28.50 ± 0.18
7	0.32	0.42	0.74	37.81 ± 0.76
2	0.18	0.6	0.78	34.41 ± 0.28
11	0.51	0.4	0.91	47.73 ± 0.52
1	0.2	0.84	1.04	41.01 ± 1.31
6	0.38	0.66	1.04	54.62 ± 0.58
10	0.54	0.62	1.16	57.20 ± 0.45
5	0.39	0.82	1.21	58.23 ± 0.05
9	0.55	0.85	1.4	61.61 ± 0.49

**Table 3:** Mechanical properties of the Al-Mg-Si alloy obtained by nanoindentation at different MAs: Nix-Gao model fitting hardness values (*H*_0_), hardness at the beginning of creep (*H*_Ini_), stress exponents of Stage I (*n*_I_) and Stage II (*n*_II_), and activation volume (V*).

MA Sample	*H*_0_ (GPa)	*H*_Ini_ (GPa)	*n*_I_	*n*_II_	V* (b^3^)
**1**	0.674 ± 0.032	0.751 ± 0.021	129.23 ± 3.21	22.26 ± 2.26	30
**2**	0.546 ± 0.002	0.608 ± 0.002	158.99 ± 2.23	18.82 ± 2.11	19
**3**	0.375 ± 0.018	0.462 ± 0.013	243.34 ± 4.19	23.14 ± 3.19	38
**4**	0.304 ± 0.016	0.424 ± 0.009	293.52 ± 3.92	34.12 ± 3.23	34
**5**	0.958 ± 0.009	1.016 ± 0.006	129.57 ± 2.57	21.23 ± 2.14	19
**6**	0.939 ± 0.015	0.993 ± 0.011	136.41 ± 1.92	48.83 ± 2.07	102
**7**	0.608 ± 0.015	0.654 ± 0.009	190.17 ± 2.17	38.65 ± 1.98	58
**8**	0.385 ± 0.016	0.495 ± 0.011	253.86 ± 2.55	57.52 ± 2.14	66
**9**	1.077 ± 0.011	1.147 ± 0.007	121.72 ± 3.01	19.53 ± 1.86	44
**10**	0.979 ± 0.012	1.081 ± 0.006	136.71 ± 2.81	74.15 ± 2.41	142
**11**	0.787 ± 0.035	0.858 ± 0.018	164.71 ± 2.13	25.56 ± 2.03	47
**12**	0.431 ± 0.014	0.504 ± 0.007	479.44 ± 3.34	68.83 ± 2.58	61

## Data Availability

The original contributions presented in this study are included in the article. Further inquiries can be directed to the corresponding author.
